# Localized Gene Delivery and Enhanced Cell–Cell Communication via Bio-orthogonal Polymer Coatings

**DOI:** 10.1007/s40883-025-00460-7

**Published:** 2025-07-25

**Authors:** Merjem Mededovic, Xiaoyang Zhong, David H. Kohn, Joerg Lahann

**Affiliations:** 1 Department of Biomedical Engineering, University of Michigan, Ann Arbor, MI 48109 USA; 2 Department of Materials Science and Engineering, University of Michigan, Ann Arbor, MI 48109 USA; 3 Biointerfaces Institute, University of Michigan, Ann Arbor, MI 48109 USA; 4 Department of Biologic and Materials Sciences, University of Michigan, Ann Arbor, MI 48109 USA; 5 Department of Chemical Engineering, University of Michigan, Ann Arbor, MI 48109 USA

**Keywords:** Gene delivery, Lentivirus, Chemical vapor deposition (CVD) polymerization, Mesenchymal stem cells (MSCs)

## Abstract

Surface modification of biomaterials, particularly by adding bioactive coatings, enhances cell-material interactions at the nanoscale, improving implant performance at the macroscale. One approach involves gene delivery via surface-bound coatings, allowing for controlled local release of viral particles. However, viral gene delivery systems, such as lentiviral vectors, face challenges in precision targeting and transduction efficiency. To address these, a bio-orthogonal coating was developed and used on titanium using chemical vapor deposition (CVD) polymerization. Co-presenting a cell binding peptide and immobilized lentiviral particles on the surface of Ti increased gene delivery efficiency by directing cells to the surface, making them easier to transduce. Specifically, a poly[(4-(3,4dibromomaleimide)-p-xylylene)-co-(4-pentafluorophenol ester-p-xylylene)] coating was prepared using CVD polymerization on Ti discs as a bio-orthogonal layer to tether lentiviral particles delivering GJA1, the gene for the gap junction protein Connexin 43 (Cx43) and the mesenchymal stem cell (MSC) binding peptide, DPIYALSWSGMA. The polymer coating exhibited high binding efficiency for both lentivirus and peptide, allowing for precise microcontact printing. Immobilized lentiviral transduction efficiency matched that in supernatant, with co-delivery increasing transduction efficiency by 35%. The biorthogonal coating boosted MSC binding 2.7-fold, leading to a density-dependent rise in cell–cell communication. High-density seeding enabled gap junction formation, while Cx43 transduction increased intercellular communication by 36%. In low-density culture, transduction led to an 84% increase in cell–cell communication within 4 h of in vitro culture. This work presents a simple, repeatable surface modification method for biomolecular immobilization, combining engineered viral vectors and peptides to enhance gene delivery approaches.

## Introduction

Surface modification of biomaterials allows for the optimization of cell-material interaction, enabling control over cellular behavior which could improve therapeutic outcomes. Surface modifications of metallic biomaterials can be classified into three categories: biological modifications, which include bioactive coatings and binding of peptides, integrins and enzymes to the surface [1]; physical modifications; and chemical modifications. Physical methods to bind biomolecules to materials, such as surface adsorption [2, 3], physical entrapment/encapsulation [3], and affinity binding [4], are effective. However, these techniques often use organic solvents, and any residual solvents can potentially be cytotoxic. Additionally, physical interactions are weak, causing unwanted or premature biomolecule dissociation and release [5]. Chemical modifications can be used to increase surface roughness for increasing cell binding or reducing bacterial growth [5], by means of acid treatments or sol–gel films, respectively [6, 7]. Biological modifications can increase cell adhesion to the implant, reduce inflammation, or increase stem cell differentiation and angiogenesis [8, 9], providing a range of tools to modulate the host response.

Binding bioactive molecules to the surface of an inorganic biomaterial with a polymeric coating is an effective strategy to maintain both molecule retention and activity. Polymer coatings prepared by chemical vapor deposition (CVD) polymerization of functional [2, 2] paracyclophane are particularly promising for the covalent immobilization of biomolecules [10, 11], as well as for targeted gene delivery [12]. Once polymerized, [2, 2] paracyclophanes result in poly-p-xylenes which offer access to a variety of side groups, and can be used to anchor biomolecules to the coating. Additionally, bio-orthogonal CVD polymer coatings offer potential for spatially controlled immobilization of multiple biomolecules [10, 11]. This spatial control allows for enhanced delivery—either spatial targeting of different molecules or co-delivery of multiple molecules to the same location. Among biomaterials, titanium stands out as an ideal substrate for CVD coating, due to its ability to withstand the temperatures in the deposition chamber and low thermal expansion which reduces the stress on the thin coatings made by CVD [13]. To immobilize multiple biomolecules onto a titanium surface, bio-orthogonal layers modified with selected functional groups have been used [14–16], such as a pentafluorophenol ester, which can directly link proteins and other biomolecules via reaction with their free amino groups [15, 16].

Bio-orthogonal coatings offer precise, controlled interaction with cells both in vitro and in vivo [17, 18]. Such control provides the opportunity to tune cell-material interaction, as well as provide a platform for understanding cell response when in contact with the material surface. Bio-orthogonal coatings can be used in a variety of applications where materials interface with cells, including orthopedic applications. For instance, bio-orthogonal coatings can deliver antibiotics to prevent infection [19, 20] as well as increase osteointegration by binding BMP2 to an implant surface [21]. The main advantage of bio-orthogonal coatings is their ability to deliver multiple peptides, drugs, and other bioactive molecules, targeting specific cells within heterogeneous populations. Bio-orthogonal coatings can also improve biocompatibility, as they are designed to be inert in vivo and in vitro while allowing for specific chemical reactions to occur. A commonly used monomer base for biorthogonal coatings [2, 2] paracyclophane is considered to be inert and does not degrade into toxic byproducts, minimizing the risk of immune activation in vivo [11].

Cell targeting is often achieved using bioactive molecules which are securely tethered to surfaces with covalent bonds. A frequently used method for achieving bioactive surfaces is tethering or eluting growth factors such as VEGF to enhance angiogenesis [2, 22] or increase migration and adhesion of osteoprogenitor cells by tethering chemotactic or cell binding peptides, such as RGD [23, 24]. Due to their small size, conjugation of peptides to a biomaterial is more precise then conjugating a full protein, and multiple chemistries can be used to conjugate the peptide to the surface [25–27]. Additionally, although RGD and IKVAV are used to bind a variety of cells, other peptides such as DPIYALSWSGMA (DPI) [28], selectively bind and home mesenchymal stem cells (MSCs), and can therefore be used for targeted cell binding [29].

Another biomaterial surface engineering application is local gene delivery. Viral gene delivery utilizes the innate mechanisms of viruses to deliver targeted genetic material into cells [30]. Viral gene delivery is still limited for in vivo uses due to off target effects and low transduction efficiency. When tethered to a metallic surface using CVD, viral delivery of BMP7 led to stimulation of osteogenic differentiation in MC3T3 cells in vitro [31]. However, optimization of the CVD coating can increase viral delivery. As opposed to delivery from a bulk material, gene delivery from the surface can be limited by material geometry. Thus, increasing the density of bound viral particles can increase the multiplicity of infection (MOI).

Lentiviruses are candidates for augmenting cell function, as they transduce both dividing and non-dividing cells and cause limited immunogenicity [32–34]. Specific to bone, lentiviruses have been used to deliver BMP2 to MSCs ex vivo [35–37], resulting in new bone formation 3 weeks post implantation of transduced cells. An alternative approach is increasing cell–cell communication by lentiviral delivery of GJA1, the gene for the gap junction protein Connexin43 (Cx43), in MSCs prior to transplantation. Cx43 is one of the most ubiquitous connexins, present in a variety of cells and is the most prevalent connexin in bone cells, being expressed in MSCs, osteoblasts, osteocytes, and osteoclasts [38, 39]. Given the ubiquity of Cx43 and its important effects in bone, Cx43 upregulation can be used as a tool to improve the function of numerous cell types critical for regenerating or healing bone. Transduction of GJA1 into MSCs led to a 1.5 × increase in the volume fraction of regenerated bone [40].

An alternative approach to Cx43 delivery is to tether lentiviral particles to a material and target host cells. However, MSCs are found in very low percentages (0.001%) of the overall heterogeneous cell population in periosteal and bone marrow tissue [41]. Therefore, the design of a coating which co-tethers a gene delivery system with a cell-targeting peptide can potentially increase the efficiency of gene delivery.

The objective of this study was to design a biomaterial coating that can upregulate cell–cell communication in MSCs via gene delivery. To address the limitations of targeted delivery to cells in a heterogeneous cell population, a novel bioactive coating was designed. A lentivirus delivering GJA1 and the MSC-homing and binding peptide, DPI, were bound to a Ti surface using a CVD co-polymer. Following validation of virus and peptide (via microcontact printing) conjugation to the surface, MSC adhesion, Cx43 transduction, and Cx43 expression were measured. Finally, cell–cell communication was measured via dye transfer assays as a function of cell density to establish the efficacy of the coating strategy to increase GJIC in MSCs. These innovations present a promising strategy to modulate cell communication and can ultimately be used for any application in which recruitment of host MSCs, followed by the upregulation of cell–cell communication and its downstream effects would boost regenerative potential. Specific orthopaedic applications include the coating of intramedullary rods to accelerate fracture healing, coating joint replacements and dental implants to increase osseointegration and meshes for tissue engineering.

## Methods

### Chemical Vapor Deposition (CVD) Polymerization

Commercially pure titanium substrates (1 cm × 1 cm) were machined from 2.54 cm × 0.05 cm (diameter × thickness) discs using a computerized numerical control (CNC) setup. The substrates were sonicated in ethanol for 5 min before and after CVD polymerization. A layer of co-polymer was deposited on the titanium substrates via a custom-built CVD system. The synthesis of 4-(3,4-dibromomaleimide)-[2.2]paracyclophane and 4-pentafluorophenyl[2.2]paracyclophane is described elsewhere [15, 42]. A 1:1 molar mixture of these two precursors was sublimated into a gas phase at 100 °C. For CVD polymerization, Ar flow was introduced into the CVD system with a 20-sccm flow rate. Activation of the precursors in the pyrolysis zone occurred at 550 °C. Subsequently, the reactive species were transferred into the deposition zone, where the Ti substrates were placed onto a rotating stage set at 15 °C. The absolute pressure of the system was controlled at 10 Pa.

### Surface Characterization via X-ray Photoelectron Spectroscopy (XPS)

XPS spectra were measured by monochromatic Axis Ultra X-ray photoelectron spectrometer (Kratos Analyticals, UK) with Al Kα X-ray source at 160 eV and 20 eV for survey and high-resolution spectra, respectively. All spectra were calibrated with a binding energy of C 1 s at 285 eV. Elemental composition was calculated based on chemical structure excluding hydrogen, for example, atomic% of O in C_6_H_5_OH is 1/(1+6)*100% (1 O in 1 O + 6 C).

### Immobilization of Anti-lentivirus Antibody

The polymer-coated Ti substrates were sterilized in 70% ethanol for 1 h after CVD polymerization. Each sample was then added to 1 ml of 10 μg/ml solution of anti-lentivirus antibody (anti-VSV-G antibody, 8G5F11 Kerafast) in phosphate-buffered saline (PBS) and incubated on a stage rotator at 4 °C. After overnight incubation, PBS was used to rinse samples 5 times (5 min each) to remove the non-immobilized primary antibody. A saturation curve over a concentration range of 0–20 μg/ml determined the use of a 10 μg/ml concentration for the antibody in subsequent experiments (see Fig. 3C in Results).

### Verification of Anti-lentivirus Antibody Binding

Samples were incubated in DPBS solution containing 10 μg/ml AlexaFluor 488 Goat antiMouse IgG secondary antibody (Invitrogen, Inc.), 0.02% (v/v) Tween20 and 0.1% (w/v) bovine serum albumin (Sigma-Aldrich) for 1 h at room temperature, followed by 5 × 5 min PBS rinses. Afterward, fluorescence microscopy (EVOS M7000) and ImageJ were used to quantify the fluorescence signals on uncoated and polymer-coated substrates with combinations of antibody and lentivirus attached. A saturation curve using a simple dilution was performed prior to exposure to cells to ensure optimal use of antibody.

### Lentivirus Design and Immobilization

Lentiviral design was completed with the assistance of University of Michigan Vector Core. The lentivirus was pseudotyped with the vesicular stomatitis virus glycoprotein G (VSV-G) to expand the tropism of the virus. The VSV-G pseudotyped lentivirus was used to deliver three genes of interest: GJA1, copGFP, an enhanced version of the GFP protein, for reporting and quantifying transduction efficiency, and puromycin for selection. As opposed to binding the copGFP to the gene of interest, GJA1, copGFP, and GJA1 were placed on two separate promoters. This was done to ensure function of the protein once Connexin43 is integrated into the cell membrane.

After immobilizing both antibody and peptide on coated Ti, the samples were incubated in lentivirus solution (3 × 10^6^ particles in 1 ml PBS) at 4 °C for 24 h. The samples were then rinsed with PBS 5 times.

### Viral Particle Visualization and Binding Validation via Scanning Electron Microscopy (SEM)

Samples were fixed in 2.5% glutaraldehyde in PBS for 30 min, then rinsed with distilled water, dehydrated with ethanol, and desiccated under vacuum overnight. SEM images were captured using a Thermo Fisher Helio G4 PFIB UXe microscope. All images were taken at 10,000 × magnification. Particles in the 70–100-nm diameter range were counted using SEM images from 5 random fields/sample.

### Immobilization of Peptide

Cysteine-functionalized DPIYALSWSGMA peptide was synthesized by the Proteomics and Peptide Synthesis Core, University of Michigan, using solid phase synthesis and protective chemistry. High-performance liquid chromatography was used to verify > 95% purity. Peptides were stored at – 20 °C until use, and dissolved in PBS at 1 mg/ml to avoid precipitation from solution. MSCs bind to DPI closer to the amine terminus of the sequence; thus, modifications to bind the peptide to the surface were done on the carboxy terminus. Due to the bioconjugation chemistries, cysteine was added to the carboxy terminus, together with two glycine spacers to form a final peptide sequence DPIYALSWSGMAGGC, which is referred to as DPI. The addition of the three amino acids insignificantly shifted the net charge (0 to − 0.05), isoelectric point (6.76 to 6.05), and hydrophilicity (− 0.67 to − 0.60) of the peptide. A FITC tagged version of the peptide was used as a control to verify peptide binding onto the surfaces, and fluorescence patterns on the sample surface were visualized after microcontact printing (μCP), while fluorescence was minimal following solution immobilization. Microcontact printing was therefore used to immobilize the peptide on antibody-attached polymer surfaces. PDMS stamps were created as described elsewhere [45].

TRITC-conjugated peptides were utilized to confirm the binding efficiency of peptides on μCP surfaces. One hundred microgram per milliliter TRITC-peptide in PBS solution was prepared as ink to print onto co-polymer-coated Ti sample surfaces for confirmation of peptide immobilization. A lower concentration was used for cell binding experiments. The peptide was immobilized onto the co-polymer coating through the rapid reaction between the thiol group and maleimides. After oxidizing for 10 min by UV-ozone, the stamps inked with peptide solution (10 μg/ml in PBS) were kept in contact with the surface of the sample for 4 h. After stamps were removed, the patterned samples were rinsed with PBS and deionized water. Peptide binding to the surface without using μCP was the same as described above.

### Validation of Peptide Binding to MSCs

The binding of MSCs to the peptide was validated using flow cytometry and TAMRA tagged DPI peptide. MSCs were stained with Hoechst 33,342, counted and split into tubes with 300,000 cells per tube. The cells were pelleted and maintained in flow cytometry buffer (PBS, 10% FBS). 10 μl of 1 mg/ml solution of fluorescently tagged peptide was added to the flow cytometry buffer for 30 min and cells were washed 2 times prior to flow cytometry.

### Cell Culture, Transduction, and Attachment to the Biomaterial

Human mesenchymal stem cells (MSCs, RoosterBio Inc) were cultured in RoosterNourish media and studies were performed on cells in passages 3 to 5. For transduction with viral supernatant, cells were seeded at 2.0–3.0 × 10^5^ cells/9.6 cm^2^ 24 h prior to transduction. On the day of transduction, viral stock solution (10^7^ cfu/ml) was added to fresh DMEM supplemented with 10% FBS and 10 μg/1 ml Polybrene (Sigma Aldrich). Cells were incubated at 37 °C for 24 h, and the media was replaced with RoosterNourish media. Imaging to quantify transduction efficiency (TE) was performed 72 h after introduction of viral vectors. Cell nuclei were stained with Hoechst 33,342 and imaged using fluorescence microscopy (Leica DMi8), observing copGFP fluorescence in cell bodies and fluorescent nuclei. To determine the optimal multiplicity of infection (MOI), a range of viral stock solution was added and TE was assessed 72 h later. The TE was determined from the fraction of copGFP-positive cells in the population, which was transduced into the cells by the lentivirus, in a region of interest of 1.2 mm^2^. The highest TE was reached with an MOI of 13, which was used in subsequent experiments. Transduction efficiency achieved with viral supernatant was utilized as a positive control, as the virus in the supernatant can bind to cells more freely.

For transduction via immobilized lentiviral particles, human mesenchymal stem cells (MSC) (RoosterBio Inc) were adhered to Ti samples co-presenting lentiviral particles and peptide, and control samples (samples with incomplete coating, lacking polymer, antibody or lentivirus) at a density of 10,000 cells per well. Each sample was placed in an individual well in a 12-well plate and incubated for 72 h in growth media (RoosterBio Inc). Live cell nuclei were stained with Hoechst 33,342 and fluorescence microscopy (EVOS M7000), and ImageJ were used to quantify the fluorescence of copGFP.

### Protein Expression

Western blotting was used to assess Cx43 upregulation 72 h after transduction. Cells were lysed with RIPA buffer. Total protein concentrations were quantified using a BCA assay and then normalized within samples for SDS-PAGE. After SDS-PAGE, electroblotting, and an overnight blocking step with 3% BSA, a Cx43 rabbit polyclonal primary antibody (Abcam) was used, followed by a goat anti-rabbit IgG HRP-linked secondary antibody (Abcam). To control for protein concentration, the membrane was also probed for β-Actin using a rabbit IgG primary antibody (CellSignaling). The membrane was imaged using ChemiDoc and analyzed using ImageLab (BioRad).

### Cell–Cell Communication Assessment—Dye Transfer Assay

A dye transfer assay was performed to quantify increases in cell–cell communication secondary to Cx43 upregulation. Donor cells were stained with a permeable dye, Calcein-AM, while the recipient cells were stained with a non-communicable membrane dye, DiL. MSCs were detached from a flask at confluence or 6-well plates if cells were transduced prior, counted, and diluted to 1 × 10^6^ cells/ml in PBS. The cell suspension was split into 2 populations at a ratio of 1:8. The smaller fraction of cells was exposed to 10 μM calcein-AM for 10 min at 37 °C and the larger fraction of cells was exposed to 5 μl of DiL per ml of cell suspension. Cells were pelleted, washed, and then seeded at a 1:8 ratio in 24 well plates, for a total of 10,000 cells per well.Three hundred microliters of media was added. For control groups, GJIC was inhibited using 100 μM of 18-α-glycyrrhetinic acid (AGA). Cells were incubated for 5 h, stained with Hoechst 33,342 then imaged using a fluorescence microscope (Leica DMi8). The fraction of cells accepting the dye was quantified by measuring the percent of recipient cells that become stained with the dye.

### Statistical Analysis

Prism 8.0 was used for data analysis. Significant differences between groups were identified via Student’s *t*-tests for transduction efficiency (Figs. 3E and 4A) and Cx43 protein levels (Fig. 4B), one-way ANOVA for viral binding (Fig. 3A), virus particle number (Fig. 3B), transduction efficiency (Figs. 3D and 6D), and cell counting (Fig. 6) or two-way ANOVA for density (Fig. 4E and F) and time dependence of dye transfer (Fig. 5).

## Results

**Fig. 1:**
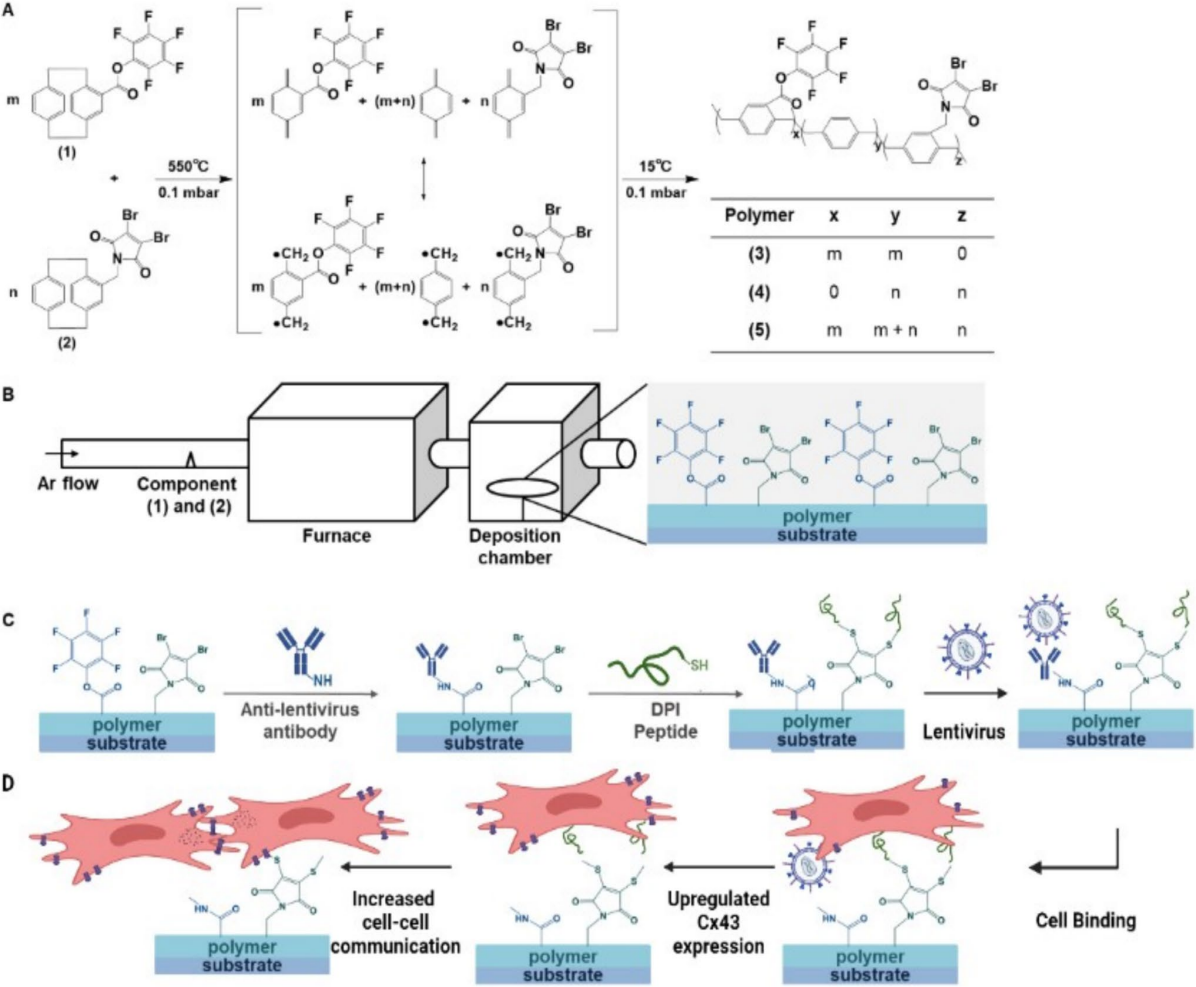
Surface engineering approach taken to functionalize titanium substrates. **A** CVD co-polymerization of [2.2]paracyclophanes with a pentafluorophenyl ester (precursor **1**) and dibromomaleimide groups (precursor **2**). **B** Co-polymerization process in a custom-made CVD system. **C** Co-immobilization of the lentivirus vector and the DPI peptide (**D**) MSC interaction with the surface coating, leading to increased expression of Cx43, and increased cell–cell communication

Titanium disks were coated using CVD to co-tether a lentivirus delivering GJA1 and an MSC-binding peptide, DPI. To achieve this, two modified [2.2]paracyclophane precursors were synthesized: one designed to selectively bind an anti-lentiviral antibody, facilitating viral tethering, and the other to anchor the MSC-binding peptide. The chemical reactions involved, along with the sequence of biomolecule binding to the surface, are shown in Fig. 1.

Both 4-pentafluorophenyl-[2.2]paracyclophane (precursor **1**) and 4-(3,4-dibromomaleimide)[2.2]paracyclophane (precursor **2**) were selected to present pentafluorophenol ester and maleimide groups on substrates. The mixed precursors **1** and **2** were co-polymerized on titanium (Ti), following the chemical reaction shown in Fig. 1A. This polymerization process was performed in a custom-made chemical vapor deposition (CVD) system (Fig. 1B). The precursors were selected due to their specific side functional groups, which can bio-orthogonally immobilize different molecules with covalent bonding. To impart MSC targeting capabilities, a cysteine spacer was added to DPI to tether it onto the substrates via reactive maleimide groups [44]. Before investigating co-polymer **5**, establishment of poly[(4-pentafluorophenyl ester-*p*xylylene)-*p*-xylylene-*co*-(*p*-xylylene)] (polymer **3**) and poly[4-(3,4-dibromomaleimide)-*co*-(*p*xylylene)] (polymer **4**), was necessary, as they each carry one of the two relevant functional groups. Thus, polymer 3 binds to the anti-lentivirus antibody via an ester reaction and polymer 4 binds to DPI via a thiol-maleimide reaction.

**Fig. 2:**
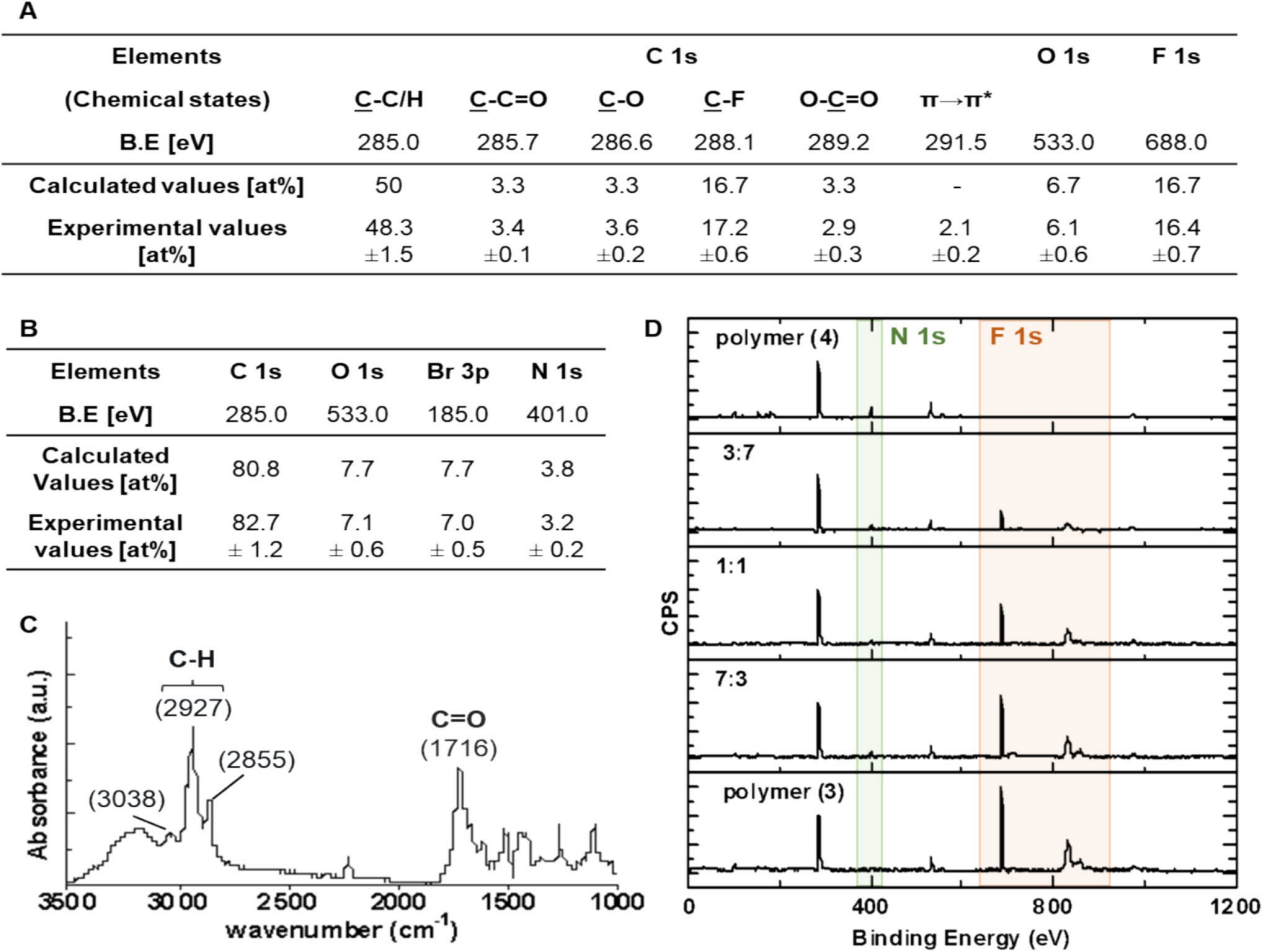
Chemical characterization of CVD samples. **A** XPS analysis of titanium disks coated with polymer **3 (**poly[(4-pentafluorophenyl ester-*p*-xylylene)-*co*-(*p*-xylylene)]**)**. **B** XPS analysis of titanium discs coated with polymer **4 (**poly[4-(3,4-dibromomaleimide)-*p*-xylylene-*co*-(*p*xylylene)]**)**. **C** FT-IR spectrum of polymer **4 (**poly[4-(3,4-dibromomaleimide)-*p*-xylylene-*co*-(*p*xylylene)]**)**. **D** XPS spectra of co-polymer with different molar ratios

The XPS data for polymer **3** (Fig. 2A) and polymer **4** (Fig. 2B) confirm the presence of the functional groups. The elemental composition of the polymers corresponds with the expected chemical composition based on its chemical formula. The XPS data were further corroborated by Fourier-transform infrared spectroscopy (FT-IR), revealing a characteristic C = O band at 1716 cm^−1^ (Fig. 2C). Moreover, the presence of symmetric and asymmetric C-H stretching modes was confirmed by the characteristic bands at 2855, 2927, and 3038 cm^−1^.

After confirming the deposition of polymers **3** and **4**, mixed precursors **1** (corresponding to polymer 3) and **2** (corresponding to polymer 4) with different molar ratios were co-polymerized onto Ti surfaces. The ratios labeled in the XPS spectra (Fig. 2D) reflect the ratios of the mole number of precursor **2** to the mole number of precursor **1**. All XPS spectra were normalized to the peak area of C_1s_ to compare the atomic percentage of each element in different ratios of the co-polymers. From top to bottom, the relative amount of precursor **2** decreased resulting in a decreasing of N_1s_ signal (representing the dibromomaleimide group) and an increasing F_1s_ signal (representing the pentafluorophenyl ester group). The ratio between different side functional groups on the surface can therefore be engineered by changing the molar ratio of precursors **1** and **2**. A 1:1 precursor ratio creating poly[4-(3,4-dibromomaleimide-p-xylylene)-co-(4pentafluorophenyl ester-p-xylylene)-co-(p-xylylene)] was used in all experiments and is defined as co-polymer coating** 5**.

Once the final co-polymer **5** was synthesized, the lentiviral particles and peptide were adhered to the coating. The lentiviral particles were bound to the surface with an anti-lentivirus antibody using antibody-antigen interaction. Anti-lentivirus antibody was immobilized via ester reaction with amines, which were carried by CVD coating and antibody, respectively (Fig. 1C).

**Fig. 3:**
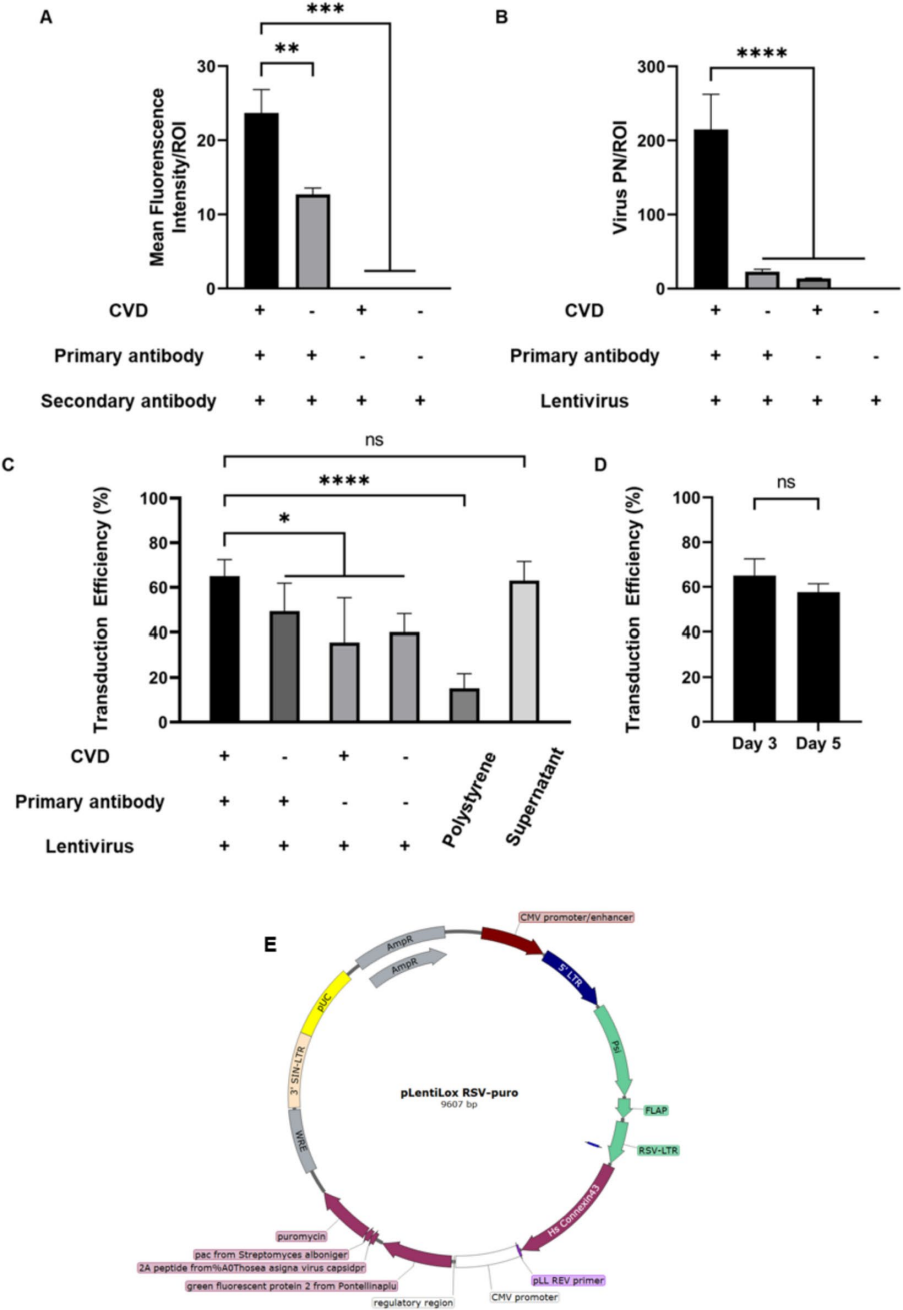
Confirmation of viral binding to bio-orthogonal coatings and transduction of MSCs. **A** Mean fluorescence intensity within the region of interest (ROI) of polymer-coated and non-coated Ti discs with/without incubation in anti-lentivirus antibody and Alexa fluor 488-conjugated secondary antibody. **B** Quantification of viral particle number (PN) on different samples by scanning electron microscope (SEM) imaging. **C** Saturation curve of antibody conjugation on CVD-coated Ti films. **D** Transduction efficiency (%) of human MSCs on different sample surfaces/solution after a 3-day incubation. **E** Transduction efficiency (%) of human MSCs on polymer-coated Ti samples with immobilized lentivirus after 3 days (left) and 5 days (right) incubation. **F** Viral plasmid used to deliver GJA1, containing copGFP for imaging and puromycin for selection. Values are reported as means ± SD, *n* = 5. ROI = 50 μm.^2^. *: < 0.05; **: < 0.005; ***: < 0.0005; ****: < 0.00005

Saturation of the CVD functional groups was observed at antibody levels above 10 μg/ml (Fig. 3C). A significantly higher mean fluorescence intensity on polymer-coated Ti was measured than on surfaces without CVD coating (Fig. 3A), confirming direct antibody binding on the polymer-coated substrates. Even though the secondary antibody also contains an amine group, under our conjugation conditions, secondary antibodies only bind with anti-lentivirus antibodies, as no fluorescence intensity was detected on CVD-coated samples that incubated in secondary antibodies alone (Fig. 3A). Significantly more lentivirus was attached to the polymer-coated Ti surface than the non-polymer substrate (Fig. 3B), confirming the binding between the anti-lentivirus antibody and lentivirus. Thus, although non-specific binding of primary antibody was observed in the absence of the reactive CVD coating (Fig. 3A), we concluded that non-specific viral immobilization in the absence of CVD coating was negligible (Fig. 3B).

CVD-coated Ti samples had significantly higher transduction efficiency (TE) than samples where one of the polymers was missing, confirming the function of CVD-based co-polymers on viral immobilization (Fig. 3D). No significant TE difference was found between supernatant and CVD-coated samples, indicating the surface modification methods did not affect the virus binding to the cells. Moreover, TE of samples incubated with lentivirus for 5 days was not different from TE after 3 days (Fig. 3E), indicating that the substrates were still functional after longer incubation.

**Fig. 4:**
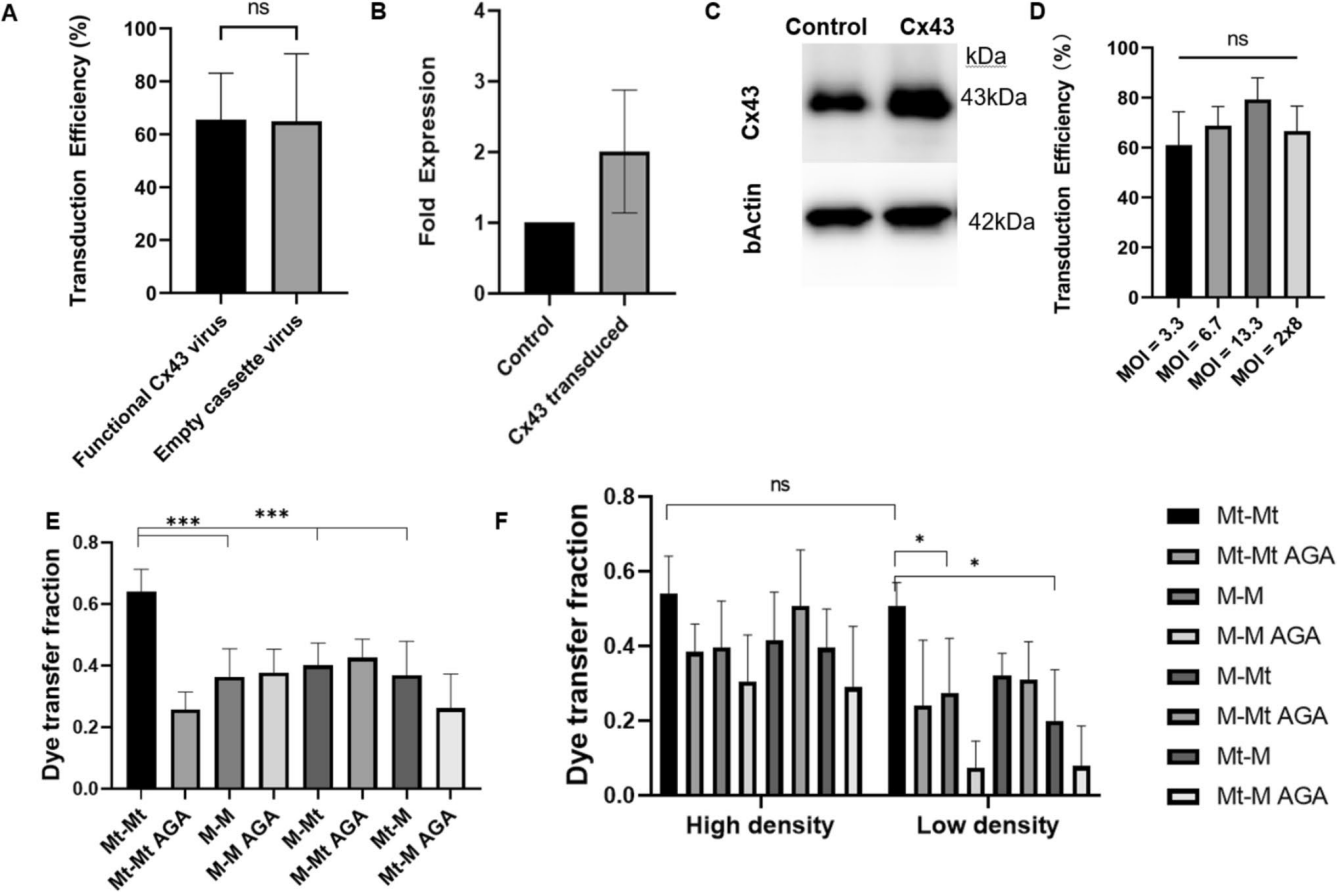
Lentiviral transduction leads to increased Cx43 expression and cell–cell communication. **A** Transduction efficiency (%) of blank and Cx43-delivering viral particles 72 h post-transduction and **B** quantified protein expression of Cx43 in transduced and non-transduced cells 72 h post-transduction. **C** Protein expression of Cx43 via Western blot in transduced and non-transduced cells 72 h post-transduction. **D** Transduction efficiency at different multiplicities of infection (MOI). **E** Cell–cell communication among transduced (Mt) and non-transduced (M) MSCs. **F** Effect of cell density on cell–cell communication (high density: 21,000 cells/cm^2^; low density: 5000 ells/cm^2^). Values are reported as means ± SD, *n* = 10. ROI = 50 μm.^2^. *: < 0.05; **: < 0.005, ***: < 0.0005

Cx43 transduction was conducted with standard stock pLLRSV GFP vector (University of Michigan Vector Core). The stock vector was used to package and deliver GJA1, the gene for Cx43 (Fig. 3F) for a final vector pLL RSV Cnx43-CMV-CopGFP-T2a-Puro. There was no significant difference in transduction efficiency as a result of gene insertion into the viral plasmid (Fig. 4A). Expression of Cx43 protein in transduced MSCs was twofold higher (2.01 ± 0.87) compared to non-transduced MSCs (Fig. 4B and C). There was also no significant difference in transduction efficiency for multiplicities of infection (MOI) ranging from 3.3 to 13.3 (Fig. 4D). Although no significant difference was found, the mean transduction efficiency exceeded the 60% threshold at MOI = 13.3, including 1 standard deviation away from the mean, which was not true for other MOIs.

A functional measure of gap junction intercellular communication, a dye transfer assay, was performed to assess whether exogenous incorporation of GJA1 and increased expression of Cx43 leads to changes in cell–cell communication. At a moderate cell density (10,500 cells/cm^2^), the dye transfer fraction was highest when both donor and recipient cells had upregulated GJA1 expression (63.9% ± 7.3%). This is a significant increase compared to the dye transfer in control MSCs (36.4% ± 9.2%) (*p* < 0.0002) and in a cell population when only the donor (Mt-M 36.9% ± 11.0%) or recipient (M-Mt 40.1% ± 7.3%) cells have upregulated GJA1 (*p* < 0.0005) and (*p* < 0.0002) respectively (Fig. 4E). The collective data in Fig. 4 demonstrate successful transduction with pLL RSV Cnx43-CMV-CopGFP-T2a-Puro, a twofold increase in Cx43 activity, and significant increases in cell–cell communication between transduced MSCs, which are density dependent (Fig. 4F), where the impact of Cx43 is more pronounced at lower cell density, where baseline cell–cell communication is lower.

**Fig. 5:**
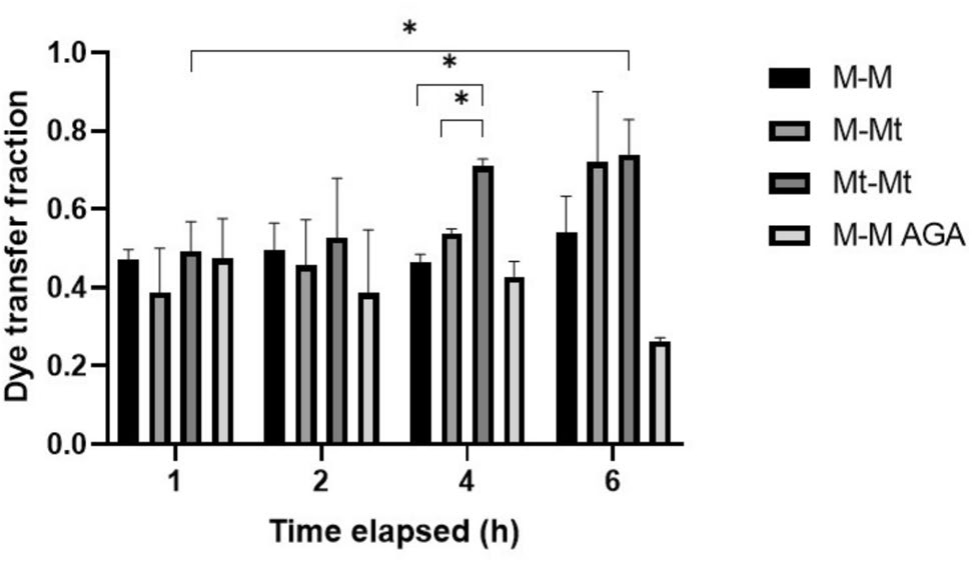
The fraction of communicating cells increases in transduced cells with time at a density of 10,000 cells/cm^2^. Values are reported as means ± SD, *n* = 10. ROI = 50 μm.^2^. *: < 0.05

Delivery of the GJA1 gene also resulted in a time-dependent increase in cell–cell communication, where the homogeneously transduced (both donor and recipient cells are transduced: Mt-Mt) group showed a significant increase in the fraction of communicating cells at 4 h, compared with both the heterogeneously transduced (only the recipient cells are transduced: M-Mt) and the control group (Fig. 5).

**Fig. 6:**
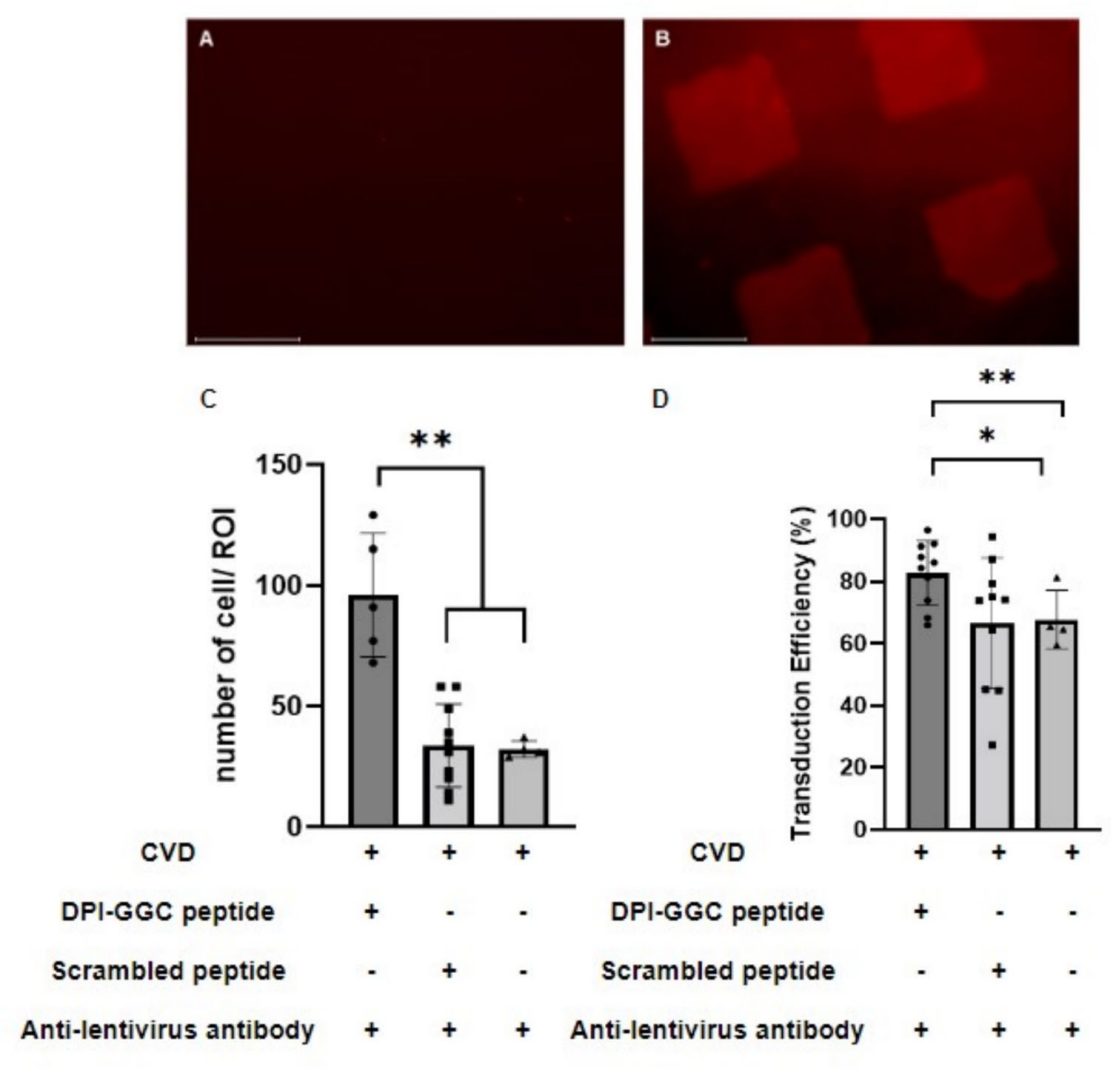
Effect of surface-immobilized MSC-binding peptide on viral transduction. **A** and **B** Comparison of peptide binding efficiency. The peptide was immobilized on polymer-coated substrates by **A** reaction in solution and **B** microcontact printing, μCp, for 4 h at room temperature, 10 × magnification. **C** Number of cells on samples with or without peptide and **D** transduction efficiency (%). Values are reported as means ± SD, *n* = 10. ROI = 50 μm^2^. *: < 0.05; **: < 0.005 Scale bar = 275 nm

To increase attachment of MSCs to the surface coating, increasing the likelihood of transduction with the immobilized viral vector, the MSC-binding peptide, DPIYALSWSGMA, was conjugated to the coating. Fluorescence of the tagged peptide confirmed peptide binding via microcontact printing (μCP), compared to simple adsorption (Fig. 6A and B respectively). The function of the MSC-binding peptide after immobilization was assessed by counting bound cells on the coated Ti surface. Flow cytometry confirmed that MSCs retained high binding to the peptide after addition of a fluorescent tag and a modification to allow for a thiol-maleimide reaction (data not shown). There was a significant 2.7-fold increase in cell binding with μCP immobilized peptide samples (Fig. 6C), confirming the MSC-binding function of DPI peptide.

Moreover, the TE increased from 60 to 82% on surfaces which had the DPI peptide immobilized via μCP, compared to a scrambled peptide (Fig. 6D).

## Discussion

Chemical vapor deposition of two modified [2.2]paracyclophane monomers was used to coat titanium for both recruiting and binding of mesenchymal stem cells and for gene delivery of GJA1. This approach provides spatial control of the two monomers and demonstrated that co-presentation of lentiviral particles with cell-homing peptides increases transduction efficiency, while also significantly increasing the number of cells transduced (Fig. 6). Overall, a material-based approach to augmenting host cell–cell communication can potentially be used to overcome the drawbacks of in vivo gene delivery, including low transduction efficiency, and delivery in heterogeneous cell niches. This strategy could be applied to a variety of clinical scenarios, including the coating of intramedullary rods to accelerate fracture healing, coating joint replacements and dental implants to increase osseointegration and coating meshes for increasing tissue regeneration.

[2.2]Paracyclophane is commonly used for immobilizing biomolecules [10, 12], as it is not cytotoxic, it is stable in physiological conditions and can be functionalized to attach various biomolecules to the surface of materials. [2.2]Paracyclophane is hydrophobic due to its benzene rings; however, its modifications can affect wettability [43]. Although hydrophobic materials can reduce cell adhesion, due to the tethering of an MSC-binding peptide, the inhibitory effect of hydrophobicity on cell adhesion was considered negligible. [2.2]Paracyclophane provides a nanoscale-thick and relatively smooth coating on metallic surfaces. Prior studies using [2.2]paracyclophane-based coatings for gene delivery using dental implants demonstrate the robustness of such coatings and their effectiveness despite friction during implantation [31].

The [2.2]paracyclophane monomers were designed to bind a lentiviral particle delivering GJA1, the gene for the gap junction protein connexon-43 and an MSC-binding peptide, DPIYALSWSGMA. The monomers were modified to present both pentafluorophenol ester, which bound the anti-lentivirus antibody via an ester reaction, and a maleimide group which bound the peptide via a thiol-maleimide reaction. Similar bio-orthogonal coatings have been used to bind BMP-2 to titanium to promote osseointegration [21] and Runx2 to poly (ε-caprolactone) [46]. However, the strategy of co-delivery of a cell-homing peptide and lentivirus can provide longer-term results, as BMP-2 therapy is known to require large doses in vivo, and is bioactive for a short period of time.

XPS analysis confirmed the presence of poly[(4-pentafluorophenyl ester-*p*-xylylene)-*co*-(*p*xylylene)], the monomer used to immobilize the lentiviral particles, (poly[4-(3,4dibromomaleimide)-*p*-xylylene-*co*-(*p*-xylylene)], used to immobilize DPI, as well as ratios of the two polymerized together (Fig. 2). Using this combination of [2.2]paracyclophane-based monomers and lentiviral particles, 200 viral particles were bound to an ROI of 50 μm^2^ (Fig. 3), which is higher or comparable to other approaches where CVD was used for only viral immobilization [45, 46]. Immobilization of the biomolecules did not impede transduction efficiency, as lentiviral transduction efficiency met the same benchmark of 60% as transduction in solution (Figs. 3D, 4A, and 6D) and led to a doubling of the expression of Cx43 protein (Fig. 4B). Increased Cx43 expression ultimately led to a 75% increase in cell–cell communication in homogenous groups of cells (both receiver and donor cells are transduced or not) (Fig. 4e). Cx43 transduction of MSCs also led to changes in cell–cell communication that were dependent on cell density (Fig. 4F) as well as time (Fig. 5). The relative change in communication between transduced and control cells is diminished at high cell density (21,000 cells/cm^2^), due to the increased baseline communication occurring in a more tightly packed cell culture (Fig. 4). The impact of GJA1is amplified at low cell density (5000 cells/cm^2^) (Fig. 4F). Lentiviral transduction also led to an earlier increase in cell–cell communication in the homogenously transduced groups, which is shown in the spike in dye transfer at 4 h, whereas the control group did not reach that level, even by 6 h (Fig. 5).

Cell homing to the material due to the peptide both increased the number of cells available to be transduced threefold (Fig. 6) as well as increased the transduction efficiency of the virus due to increased cell binding. This synergy was observed between the bound viral particles and DPI peptide, where the transduction efficiency increased ~ 33%, even though the number of cells bound to the surface increased threefold. The results indicate that with stronger cell binding to the material, the transduction capacity of a material increased ~ fourfold (Fig. 6). The synergy is likely due to the stronger bond between cell and material surface and the chemotactic effect of the peptide.

Co-immobilization of a lentiviral particle and a cell-homing peptide increased efficiency of gene delivery by increasing transduction efficiency. MSCs reside in the bone marrow, which is a heterogeneous cell niche. Thus, preferential binding of MSCs to the material surface can increase the contact between the target cell population and the immobilized viral vectors. This co-immobilization approach potentially reduces some of the drawbacks of lentiviral gene therapy, poor transduction efficiency and off-target effects, broadening its utility. The efficiency of gene delivery of viral particles can be increased by co-delivering viral particles with a peptide that homes and binds MSCs, as presented in this paper. Immobilization of the lentiviral particles allows for local gene delivery, as opposed to systemic in vivo gene delivery, while co-immobilization with a cell-homing peptide can provide targeted delivery within a diverse cell population. Collectively, these two strategies can potentially increase transduction in vivo.

CVD coating of [2.2]paracyclophane allows for a straightforward modification of the spatial distance between monomers by changing the molar ratio of the two precursor monomers. Although only one ratio (1:1) was used in this study, the use of [2.2]paracyclophane-based monomers provides the opportunity for optimization of the ratio of viral particle to peptide on the surface.

The ratio of precursors can be altered to find an optimal balance between number of cells and cell transduction that would maximize cell–cell communication. An optimal coating would provide the highest MOI while maintaining high specificity for MSCs. Additionally, modifying the dimensions of the PDMS stamp can spatially control peptide patterning on the polymeric coating. Such spatial patterning may more effectively direct the MSC migration towards repair sites, as opposed to uniformly along the implant surface itself.

Our results show that co-immobilizing a peptide with a gene vector increases transduction efficiency by ~ 33% (Fig. 6). This approach could amplify the effects generated by the virus alone. As an example, lentiviral particles with an MOI of 200PFU/cell led to a 3.5-fold increase in Cx43 expression and increased MSC osteo-differentiation as demonstrated by a fourfold increase in osteocalcin mRNA expression and a fivefold increase in BMP2 mRNA expression, [40]. The data presented in the current study shows a doubling of Cx43 with a much smaller MOI of 13.3, demonstrating an opportunity to produce safer and more effective viral gene delivery therapy.

Ultimately, in vivo studies would confirm the effectiveness of upregulating cell–cell communication in MSCs in a heterogeneous cell niche, and its potential therapeutic effects. While orthopedic implants are designed for long-term use, the surface coating and its bioactive components are intended to function only during the initial phases of bone fracture healing or implant integration. Future work involves coating biomaterials used to stabilize bone fractures to determine the osteogenic effects of upregulated cell–cell communication in vivo. Animal studies would also assess the impact of material design. These studies can confirm the ability of the coating to catalyze in vivo transduction and increase transduction efficiency when employed in concert with the cell-targeting ability of DPI.

Co-immobilizing a cell-homing peptide together with a therapeutic can be used to target other cells in heterogeneous cell populations, such as cancers or bone marrow. This strategy is also amenable to a variety of other dual-use materials, such as the addition of multiple peptides to trigger osseointegration or anti-microbial/pro-regenerative therapies and combinations of gene therapies.

## Conclusions

A CVD polymerized coating on titanium was used to co-present immobilized lentiviral particles in combination with a cell-homing peptide. The CVD coating process allows modulation of the ratio of monomers binding the lentiviral particle and peptide, providing control of concentrations and spatial control through patterning. Coordinated binding of lentiviral particles and peptides to a titanium surface using a [2.2]paracyclophane-based coating increased both cell binding by the peptide and transduction by the lentivirus—the number of MSCs attaching to the surface increased threefold, and transduction efficiencies were greater than 60%, comparable to values generated with lentiviral particles in suspension. Increasing transduction efficiency resulted in a twofold increase in Cx43 expression and a 1.8-fold increase in cell–cell communication. Although DPI is used to increase cell homing and binding from a heterogeneous cell population, the TE of the virus increases when DPI is added to the surface, allowing for a lower multiplicity of infection (MOI). These results demonstrate the potential of biorthogonal CVD coatings for biomedical applications that enable co-immobilization of viral nanoparticles and cell binding peptides or, more generally, different classes of biological entities.

## Data Availability

Data supporting the findings of this study are deposited on the University of Michigan Advanced Research Computing highperformance computing cluster. Researchers desiring access to data should send a request to the corresponding author.

## Abbreviations

CVD: Chemical vapor deposition
GJIC: Gap junction intracellular communication
MSC: Mesenchymal stem cells
XPS: X-ray spectroscopy
FT-IR: Fourier-transform infrared spectroscopy
TE: Transduction efficiency
copGFP: Green fluorescent protein from copepod
ROI: Region of interest
μCP: Microcontact printing
PBS: Phosphate-buffered saline
MOI: Multiplicity of infection
